# A decade of progress and innovation in dermatology

**DOI:** 10.3389/fmed.2024.1546471

**Published:** 2025-01-22

**Authors:** Robert Gniadecki

**Affiliations:** Division of Dermatology, University of Alberta, Edmonton, AB, Canada

**Keywords:** skin diseases, dermatology < specialty < medicine, photodermatology, inflammation, therapy, skin of color

## Introduction

The last decade has seen unprecedented advancements in dermatology, driven by progress in therapeutics, technology, and a deeper understanding of the societal needs of diverse populations. This review highlights significant milestones, recognizing that the choices are subjective and reflect achievements with a lasting impact. Key themes include therapeutic breakthroughs, innovations in skin cancer management, integration of artificial intelligence (AI), applications of quantum medicine, and the focus on dermatology for skin of color. Together, these developments underscore dermatology's evolution from a visual, descriptive specialty dominated by empirical treatments into a technologically advanced specialty anchored in general medicine (Table 1).

**Table 1:** Dermatology's decade: triumphs and challenges.

**Milestones achieved during the last decade**	**Challenges remaining**
Unprecedented therapeutic efficacy in chronic inflammatory skin diseases (e.g., psoriasis, atopic dermatitis, pemphigus) with advancements in anti-cytokine antibodies (anti-IL-23, IL-17, IL-36), JAK inhibitors, and B-cell depletion therapies (anti-CD20)	True disease modification or cure remains elusive, with most patients requiring indefinite therapy. The incidence of chronic inflammatory skin diseases continues to rise globally, driven by poorly understood environmental and lifestyle factors
Neoadjuvant therapy for invasive malignant melanoma has achieved long-term survival rates exceeding 70%, transforming outcomes in resectable cases	The causes of the rising incidence of malignant melanoma remain unclear, with environmental, genetic, and behavioral contributions yet to be fully elucidated
Artificial intelligence (AI) systems capable of accurately classifying most skin diseases based on lesion morphology have demonstrated diagnostic accuracy rivaling dermatologists	AI implementation remains limited due to legal, ethical, and regulatory concerns, as well as insufficient integration into clinical workflows. AI-driven diagnostic tools require further refinement and clinician acceptance
Recognition of the health benefits of full-spectrum light exposure (ultraviolet, visible light, infrared), including its roles in vitamin D synthesis, immune modulation, and potential tissue repair through photobiomodulation	Photobiomodulation remains underutilized, with limited technological integration and a lack of standardized protocols. Awareness of balanced UV exposure for health benefits is still lacking in public health guidelines
Widespread adoption of equity, diversity, and inclusion (EDI) principles has led to greater recognition of skin diseases relevant to populations with skin of color	Dermatology in underdeveloped countries remains underfunded and under-researched, leaving significant gaps in addressing infectious and neglected skin diseases that dominate morbidity and mortality in these regions

## Skin inflammation: from partial control to full disease remission

Dermatology has undergone a paradigm shift from symptom management to achieving durable remission and broader systemic health benefits. Psoriasis treatment, in particular, has been revolutionized by the advent of monoclonal antibodies that specifically target key cytokines such as IL-17, IL-23, and IL-36 (1). These biologic therapies have demonstrated unprecedented rates of skin clearance alongside robust safety profiles. Remarkably, addressing psoriasis-related inflammation with monoclonal antibodies has shown additional systemic benefits, including improved overall survival. This effect is likely mediated by the reduction of systemic inflammation and cardiovascular comorbidities (2, 3). These findings are transformative, suggesting that the advantages of treating chronic inflammatory skin diseases extend far beyond visible skin improvement, reshaping our understanding of the systemic impact of effective dermatologic care.

The success of biologic therapies in psoriasis has catalyzed the development of similar treatments for other chronic inflammatory diseases, including atopic dermatitis, hidradenitis suppurativa, lupus erythematosus, vitiligo, alopecia areata, and pyoderma gangrenosum, among others. Among these, the advancements in atopic dermatitis have been particularly notable. The pathogenesis of atopic dermatitis, a condition with the literal meaning “out-of-place inflammation,” was historically poorly understood. The discovery of the central role of the Th2 signaling axis has been transformative for drug development. Targeting cytokines such as IL-4, IL-13, and Il-31 as well as their downstream signaling via the JAK-STAT pathway, has resulted in sustained symptom relief (4). Patients have experienced significant improvements in pruritus, sleep quality, and psychological wellbeing, marking a new era in the management of this challenging disease. However, it remains to be seen whether these treatments will yield comparable long-term systemic benefits, as observed with biologics in psoriasis.

Interestingly, targeting soluble cytokines in skin inflammation has proven to be a more successful therapeutic approach than directly targeting immune cells in many conditions. This may be explained, in part, by the proposed hypothesis that the patterning of skin diseases—manifesting as dots, stripes, patches, or rings—is driven by the lateral diffusion and interactions of inflammatory mediators, creating two-dimensional structures (5). However, in certain autoimmune diseases, targeting immune cells has demonstrated superior efficacy. For example, in autoimmune blistering diseases, the depletion of CD20-expressing B cells using monoclonal antibodies has established new therapeutic benchmarks, achieving remission in the majority of patients (6).

## A shift in survival outcomes in malignant melanoma

Skin cancer management, particularly for melanoma, has undergone transformative advancements with the introduction of immune checkpoint inhibitors, including anti-PD-1, anti-PD-L1, and anti-CTLA-4 therapies. Melanoma, a highly immunogenic and heavily mutated tumor, ranks among the cancers most responsive to systemic immunotherapy. Traditionally, the standard treatment for resectable invasive melanoma has been surgical excision, often followed by adjuvant systemic therapy in cases of metastatic disease. However, recent long-term data on neoadjuvant combination immunotherapy (anti-PD-1 combined with anti-CTLA-4) have demonstrated significant improvements in survival rates for stage III melanoma (7). These findings, derived from studies conducted over the past decade, suggest a potential shift in treatment paradigms. In the future, patients with invasive malignant melanoma may be routinely offered neoadjuvant immunotherapy before surgical resection, achieving enhanced survival outcomes and redefining the management of this aggressive cancer.

## Expansion of artificial intelligence in dermatology

Artificial intelligence (AI) has emerged as a transformative tool in dermatology, particularly for diagnostics. Machine learning algorithms trained on large datasets of dermoscopic images have demonstrated diagnostic accuracy comparable to or exceeding that of dermatologists, particularly for melanoma and non-melanoma skin cancers (8). These systems have improved early detection and reduced diagnostic errors, enabling clinicians to prioritize high-risk cases.

AI's applications extend beyond diagnostics. Teledermatology platforms incorporating AI have enhanced access to care, particularly in underserved areas. During the COVID-19 pandemic, these technologies played a crucial role in maintaining patient care, streamlining triage, and facilitating remote consultations. AI is also being integrated into research, where it accelerates the analysis of complex datasets and supports drug discovery.

Despite its promise, the adoption of AI in dermatology raises ethical and regulatory challenges, including ensuring unbiased algorithms and safeguarding patient privacy. Addressing these issues will be essential as AI becomes more deeply integrated into clinical practice.

## Embracing quantum medicine: advances in light-based therapies

Quantum medicine, which investigates the interaction of photons with biological systems, has opened new frontiers in dermatology. Emerging research has highlighted the skin's ability to respond to non-ultraviolet light, such as visible spectrum and near-infrared wavelengths. The skin contains opsins—visible light photoreceptors also found in the retina and hypothalamus—where they play roles in regulating circadian rhythms, metabolism, and thermogenesis (9, 10). Red and near-infrared wavelengths, which are abundant in the environment due to their limited absorption by plant chlorophyll, have been shown to penetrate deeply into the skin. These wavelengths enhance mitochondrial function and promote tissue repair, providing a basis for photobiomodulation as a therapeutic approach. This has opened new avenues for managing conditions such as wound healing and inflammatory skin diseases.

The role of ultraviolet radiation (UVR) has also been re-evaluated, with increasing recognition of its dual effects (11). While UVR is a well-established carcinogen, it is also essential for vitamin D synthesis, immune modulation, and the prevention of autoimmune diseases. Epidemiological studies have shown higher prevalence rates of autoimmune disorders in regions farther from the equator, suggesting the protective effects of UVR exposure. Moreover, UVR has been inversely associated with cardiovascular events and type 2 diabetes mellitus, particularly in women (12). These findings underscore the need for a nuanced understanding of UVR's risks and benefits. Future research should focus on revisiting current recommendations of total UVR avoidance, aiming to achieve a balance that maximizes its health benefits while minimizing harmful effects.

## Responding to social needs: dermatology for skin of color

Addressing disparities in healthcare has become a significant focus in recent years, with dermatology playing a central role in the equity, diversity, and inclusion (EDI) movement. Efforts to reduce these disparities have included the development of culturally competent educational materials, targeted outreach programs, and inclusive dermatologic curricula. Several universities have established endowed chairs dedicated to advancing the dermatology of skin of color, further institutionalizing this commitment. These initiatives, often informed by principles from critical race theory, underscore dermatology's evolving responsibility in promoting equity and ensuring that advancements in research and clinical practice benefit all populations. As the importance of the EDI perspective continues to grow, it is expected to shape research priorities and influence the future of clinical care in dermatology.

## Unmet needs and directions for the next decade: toward healthy skin for everyone

One of the most pressing challenges in dermatology remains the lack of curative therapies for chronic inflammatory skin diseases. Despite significant therapeutic advancements, the global incidence of skin cancers and chronic inflammatory conditions continues to rise, highlighting an urgent need for deeper understanding. The causes behind this paradox remain elusive but are thought to include environmental and lifestyle factors such as the proliferation of processed foods, unbalanced diets, limited access to natural sunlight, and other consequences of modern living. Addressing these root causes, which often overlap with those of other chronic diseases, represents a critical frontier for future research. Investigating the shared mechanisms underlying systemic inflammation and skin diseases could lead to preventive interventions that enhance overall health, with benefits extending far beyond dermatology.

Historical perspectives also reveal how patterns of skin diseases have shifted dramatically over the past century. A hundred years ago, infections and infestations such as tuberculosis, syphilis, and scabies dominated dermatologic practice in industrialized nations. Today, these conditions are rare in developed countries but remain a leading cause of skin-related morbidity and mortality in less developed regions. Unfortunately, these infectious skin diseases receive relatively little research attention, leaving a significant gap in addressing global health disparities.

Another unmet need lies in the integration of artificial intelligence (AI) into dermatologic practice. As highlighted earlier, AI systems have demonstrated enormous potential in diagnostics and care optimization, yet their adoption has been limited by legal, ethical, and privacy concerns. The next decade should prioritize establishing frameworks that ensure the responsible and equitable use of AI while safeguarding patient confidentiality. AI has the capacity to revolutionize access to care, particularly in regions where medical dermatology services are scarce. By enabling entry-level consultations and triage, AI could alleviate bottlenecks and provide timely care for underserved populations.

With careful planning and focused efforts, the next decade holds the promise of addressing longstanding gaps in dermatology and advancing toward the goal of healthy skin for everyone.
